# Evaluation of Functional Magnetic Resonance Imaging under Artificial Intelligence Algorithm on Plan-Do-Check-Action Home Nursing for Patients with Diabetic Nephropathy

**DOI:** 10.1155/2022/9882532

**Published:** 2022-03-25

**Authors:** Qianqian Du, Dianchao Liang, Lixin Zhang, Guoyan Chen, Xueyan Li

**Affiliations:** ^1^Department of Endocrinology, the Second Hospital of Shijiazhuang, Shijiazhuang 050000, Hebei, China; ^2^Community Health Service Centre of Zhentou, Shijiazhuang 050000, Hebei, China; ^3^Department of Nursing, the Second Hospital of Shijiazhuang, Shijiazhuang 050000, Hebei, China; ^4^Department of Nephrology, the Second Hospital of Shijiazhuang, Shijiazhuang 050000, Hebei, China; ^5^Department of Peripheral Vascular Surgery, the Second Hospital of Shijiazhuang, Shijiazhuang 050000, Hebei, China

## Abstract

This study aimed to evaluate the effect of functional magnetic resonance imaging (fMRI) under the fuzzy C-means (FCM) clustering algorithm on plan-do-check-action (PDCA) home nursing for patients with diabetic nephropathy (DN). As the characteristics of fMRI image data were combined, the FCM algorithm was improved and applied into the clustering processing of fMRI activation regions of patients. 64 patients with DN were chosen as the research objects and were divided into the research group with PDCA home nursing and the control group with routine home nursing. The patients were randomly divided into the research group (*n* = 32) and the control group (*n* = 32). The curative effect, nursing satisfaction, and quality of life of patients after nursing were compared. The results showed that the coverage of fMRI activation points was significantly higher as being detected by the FCM algorithm, and the running time was shortened by 33.6 min. After nursing, the total effective rates in the research group and the control group were 87.5% vs. 34.4% in 3 months, 93.8% vs. 68.8% in 6 months, and 96.9% vs. 75.0% in 12 months, respectively; those in the research group were significantly higher than those in the control group (*P* < 0.05). The nursing satisfaction score (91.3 ± 4.5 vs. 80.9 ± 5.2) and nursing service quality score (89.7 ± 6.6 vs. 80.3 ± 7.1) in the research group were also significantly higher than those in the control group (*P* < 0.05). Meanwhile, the scores of each item after nursing in the research group were significantly higher than those in the control group (*P* < 0.05). The improved FCM algorithm detected the activation regions in the fMRI images more effectively, which could provide help for diagnosis and reduce error and misdiagnosis. At the same time, the PDCA home nursing also offered great help to the recovery of patients with DN, which was more superior for the curative effect of hospitalization, the promotion of recovery, and the improvement of patients' quality of life.

## 1. Introduction

Diabetic nephropathy (DN) is a kind of common diabetic complication. Pathologically, it is mainly observed in patients with glomerular sclerosis caused by microvascular disease, so it is also called diabetic glomerulosclerosis [1]. Statistics show that in recent years, the incidence of DN in China was increasing year by year, the prevalence has reached more than 17%, and the mortality of patients with uremia due to DN has also reached 30% or so [2]. More and more studies have proved that diabetes is one of the risk factors for end-stage kidney disease, and the current clinical methods for DN treatment are mostly to reduce blood urea nitrogen (BUN) and serum creatinine (Cr), which ignore nursing in the recovery [3]. Because DN is a type of chronic recurrent disease, only nursing during hospitalization cannot stabilize the patients' condition effectively in a long term. Therefore, an effective home nursing is of great significance to consolidate the curative effect, reduce the recurrence, and increase the quality of life of DN patients [4, 5]. The existing home nursing methods have the limitation without a flexible nursing plan, making the personalized nursing cannot be carried out depending on the characteristics of patients and specific diseases, so they have not achieved outstanding nursing advantages [6]. Plan-do-check-action (PDCA) nursing is a new kind of home nursing containing four parts, which are just the plan, do, check, and action [7]. Studies have shown that after PDCA home nursing, the clinical symptoms of patients can be significantly improved, and compared with other nursing methods, it is easier to be accepted by patients and their families [8].

Artificial intelligence technology brings new opportunities for the diagnosis of diseases. The diagnostic expert system developed on artificial intelligence technology is a program system that simulates the thinking process of medical experts in diagnosing diseases. The expert system has the ability to collect, organize, and record expert knowledge, so as to give medical advice. Besides, artificial intelligence is applied to the image processing of functional magnetic resonance imaging (fMRI) of diabetic patients, which can help doctors reduce misdiagnosis. The artificial intelligence-based diagnostic expert system not only reduces the computational time and cost of disease diagnosis but also improves the accuracy of disease classification. On the other hand, effective renal function monitoring and prognostic evaluation are also important for prolonging the survival time of patients. With the development of imaging technology, fMRI provides the possibility to monitor renal function and evaluate the prognosis of patients at the same time [9]. When fMRI is applied for the diagnosis and evaluation of the curative effect, the structures and functions are often assessed by parameters such as time series signals. However, in practical applications, the detected signals will be interfered by noise signals [10]. Therefore, it is very important to extract useful characteristic signals from the complex mixed signals. With the rapid development of computer science and technology, the intelligent algorithms can handle the difficult issues in various fields and provide new ideas for solving the issues of disease diagnosis and treatment in the medical field [11].

DN patients were taken as the research objects in this study, and the infinite norm algorithm (ICA) was introduced to scan and segment the fMRI images, so as to deal with the noise signal interference in fMRI images. Through observing and comparing differences in different imaging values in the clinical prognosis, it was expected to find a way for the evaluation of clinical prognosis of DN patients. Thereby, disease monitoring methods could be enriched to fill the gaps in this research field, and more importantly, help for clinical treatment could be offered to benefit the overall DN patients.

## 2. Methodology

### 2.1. Research Objects and Grouping

In this study, 64 patients with DN in hospital from January 2018 to December 2020 were selected as the research objects, including 38 males (59.4%) and 26 females (40.6%). Their average age was 46.37 ± 6.63 years old, and the average course of disease was 7.97 ± 3.73 years. The patients were randomly divided into the research group and the control group, with 32 cases in each group. The clinical data of patients in both groups were collected. After comparison, there was no significant difference in the average age, gender ratio, average course of disease, treatment method, renal function on discharge, and total score of SF-36 quality of life scale between the two groups (*P* > 0.05). Therefore, the two groups went with comparability. The study had been approved by the ethics committee of hospital, and the patients included and their families signed the informed consent forms.

Inclusion criteria: all objects met the Diagnostic Criteria and Staging Criteria for Diabetic Nephropathy formulated with reference to the Second National Academic Conference on Diabetes in 1999 and the diagnostic criteria of the World Health Organization. When the patients were discharged, their serum Cr and BUN were not higher than the twice of the normal levels, with mild clinical symptoms, and the total quality of life score <50 points. The total score of the patients' 36-item short form (SF-36) quality of life scale was not higher than 50 points. The patients did not have other somatic diseases, but with complete clinical data. The patients who did not meet the requirements were excluded. Exclusion criteria: the patients had other somatic diseases.

### 2.2. fMRI Examinations

3.0 T MRI equipment was used for examinations. When scanning of diffusion weighted imaging (DWI) was performed, the echo sequence of the spin-echo diffusion weighted plane was used for imaging. The time of repetition (TR) was 3000 ms, the time of echo (TE) was 60.5 ms, the field of view (FOV) was 256 × 256 mm^2^, the layer thickness was 3 mm, and the flip angle was 90°. The patients were asked to fast for at least 6 hours the day before the examination and took the supine position for abdominal scanning in the examination. The scanning included those of T2-weighted imaging (T2WI) turbo field echo (TFE) sequence in the transverse and coronal positions. After the raw data were transmitted to the workstation, the Funtool was used to process and analyze the images.

### 2.3. Improvement of Fuzzy C-Means (FCM) Clustering Algorithm

With the characteristics of fMRI data signals, the HCC distance measurement method was obviously superior to the traditional Euclidean distance method. The HCC method was implemented on the Pearson correlation coefficient and could be described as

(1) $$\begin{array}{c} D\left(x_{j},b_{i}\right)={\left(\frac{1-p_{ij}}{1+p_{ij}}\right)}^{\beta }, \end{array}$$

where *p*_*ij*_ is the Pearson correlation coefficient between the voxel point *x*_*j*_ and the central voxel point *b*_*i*_, and *β* is the penalty factor, which could be set to 1.

Since the distance from the voxel point to the central voxel point was not a simple spatial distance, further improvements needed to be made on the basis of the HCC method. A function similar to the normal distribution was constructed for distance measurement and described as

(2) $$\begin{array}{c} Dd\left(x_{j},b_{i}\right)=\left(1-p_{ij}\right)\times \;\exp\left(-\alpha \times p_{ij}\right), \end{array}$$

where *α* is the balance coefficient of the balance distance measurement function.

It was assumed that the fuzzy membership matrix in the FCM algorithm was *A*={*a*_*ij*_*|*1 ≤ *i* ≤ *n*,  1 ≤ *j* ≤ *m*}, where *n* is the number of categories, the VoxEL dataset was *X*={*x*_1_, *x*_2_,…, *x*_*n*_}, and the mean value matrix was *C*={*v*_*i*_*|*1 ≤ *i* ≤ *n*}. At this time, the target equation of the improved FCM algorithm is

(3) $$\begin{array}{c} E_{\text{FCM}}\left(X,A,C\right)=\overset{n}{\underset{i=1}{\sum }}\overset{m}{\underset{j=1}{\sum }}u_{ij}^{z}Dd^{2}\left(x_{j},b_{i}\right). \end{array}$$

0 ≤ *u*_*ij*_ ≤ 1 and ∑_*i*=1_^*n*^*u*_*ij*_=1 are assumed, and then, *D*  *d*(*x*_*j*_, *b*_*i*_) is the new distance measurement.

Finally, the coverage (accuracy) and running time were used to evaluate the algorithm. The coverage was the ratio of the sum number of correctly detected activated voxel points and nonactivated voxel points to the number of all voxel points in the measured region of fMRI images. The larger the ratio, the more accurate the regional function activation.

### 2.4. PDCA Home Nursing

After discharge from hospital, patients in the research group and the control group were required to take kidney-protecting drugs and hypoglycemic drugs routinely. Basic information such as gender, age, renal function on discharge, blood glucose, and accompanied symptoms were recorded to construct the health file of patients. The patients in the control group were treated with routine home nursing, including the telephone follow-up once a month, diet instruction, medication, exercise, rest, and follow-up visits specifically.

In addition to routine home nursing, patients in the research group were visited by specially trained full-time nurses in the follow-ups and received PDCA home nursing. According to the patients' health status, diet, nutritional level, living habits, cognitive status, and follow-up awareness, the targeted nursing plan was designed to standardize the treatment and health behaviors of patients. At the same time, the medication behavior of patients was also standardized, as a light, easy-to-digest, and high-vitamin diet was given, together with guided rest and work to avoid over fatigue. When patients suffered from the long-term, repetitive, and costly diseases, they had mental pressures, which required professional personnel to give timely psychological consultation to eliminate their anxiety, depression, and other unhealthy emotions, and help them build confidence in recovery. A home support model was established to help patients and their family members understand relevant knowledge of the disease and guide to establish communication between the family members and patients. Thus, the patients' physical and mental health could be maintained, their mental stress could be alleviated, and their ability to adapt to life was improved, thereby the compliance of the patients could be improved as well. At the first follow-up visit, relevant materials were given out to patients and their families, and the general information of the disease were introduced, making patients and their families understand the purpose, content, and expected outcome of this study. They were also guided in the implementation of the nursing plan. At the single-month follow-up, the nursing plan was revised and improved according to the issues encountered in the last month. At the even-month follow-up, the implementation effects of the nursing plan and the issues encountered were fully known and checked through the communication with the patients and their families, and the issues were then taken as the focus of nursing in the next month. At the last follow-up, the patients and their families were convened for a symposium to strengthen health education and other related knowledge, and they made evaluations on the effect of the implementation, recovery, and nursing satisfaction. The recurring and neglected issues during nursing for the patients were learned via follow-ups, and the optimal nursing plan was worked out under negotiation with patients and their family members. With the specific issues existing in implementation, the nursing plan was revised and improved, which was then transferred into the next PDCA nursing plan. After each cycle, the nursing plan became more complete and more comprehensive, so that the nursing effect will be more significant. The PDCA home nursing needed to be implemented continuously for one year to find a more effective home nursing model as well as the better effect.

### 2.5. Evaluation of Nursing Effect

3 months, 6 months, and 12 months after nursing, the patients underwent renal function reexamination, and the improvements of the patients' clinical symptoms were recorded. The evaluation was mainly completed by fully trained personnel and was carried out according to the following criteria. If it was evaluated to be remarkedly effective, all the clinical symptoms disappeared, and the level of urinary albumin excretion returned to be normal or decreased by 50% or more. Meanwhile, the blood glucose and glycosylated hemoglobin level returned to be normal or decreased by 35% or more, and the 24 h quantitative level of urine protein decreased by 50% or more, with the normal renal function indicators. If it was evaluated as effective, the clinical symptoms were significantly improved. Urine albumin excretion, blood glucose, and glycosylated hemoglobin level were all reduced, but did not reach the standard of remarkedly effective. 24 h quantitative level of urine protein, with normal renal function indicators, also did not drop down by 50% compared with that before treatment. If it was evaluated to be ineffective, there was no improvement, but even an aggravation was found on the clinical symptoms; other laboratory indicators had not changed or increased.

12 months after nursing, the SF-36 scale developed by the U S. Medical Outcomes Study was adopted to evaluate the patients' quality of life [12]. The SF-36 scale contained 36 items, which could be classified into 8 dimensions of physical functioning, role-physical, role-emotional, bodily pain, vitality, social functioning, mental health, and general health. The total score of the scale was 100 points. The higher the score, the better the quality of life of patients.

### 2.6. Statistical Analysis

SPSS 19.0 was applied for statistical analysis. The enumeration data were expressed by frequency (%), and the difference was compared under the *χ*^2^ test. The measurement data were expressed by mean ± standard deviation (x(_) ±*s*), and the independent sample *t*-test was used for the difference comparison. When *P* < 0.05, the difference between groups was statistically significant.

## 3. Results

### 3.1. Basic Data Comparison of Patients

The basic data when the patients were discharged from the hospital were compared between the research group and the control group, and the results are given in Table 1. It was suggested that there was no significant difference between the two groups in the patients' average age, gender ratio, and body mass index (*P* > 0.05). In the laboratory examination indicators on discharge, there was also no significant difference between the two groups in hemoglobin, glycosylated hemoglobin, Cr, BUN, estimated glomerular filtration rate (eGFR), and urine protein by creatinine ratio of patients (*P* > 0.05). Therefore, the subsequent results are comparable.

### 3.2. fMRI Data Analysis under Improved FCM Algorithm

**Figure 1:**
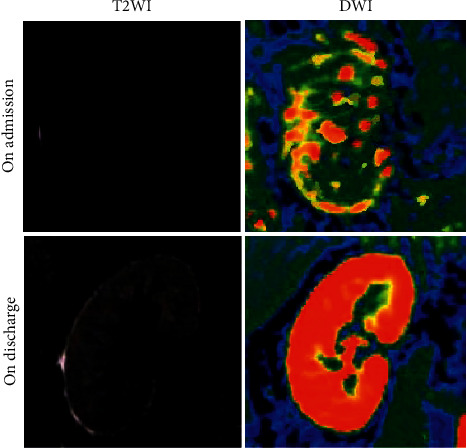
Changes in fMRI images of the patient's kidneys on admission and discharge.

fMRI was used to examine the changes in the morphology of the patients' kidney structure before and after discharge. From the images of T2WI and DWI in Figure 1, it could be observed that compared to those on admission, the kidney morphology of the patient was fuller, and the boundary of cortex and medulla was very clear on discharge. The degree of fibrosis was low in the external kidney, and the diffusion of water molecules was very even.

**Figure 2:**
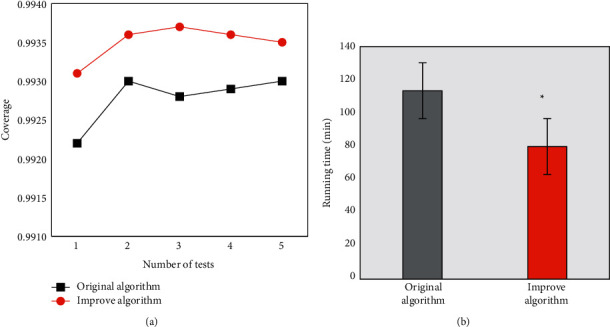
The processed results of fMRI data before and after the algorithm improvement. (a) The coverage comparison under different test times. (b) Comparison of the average running time.  ^*∗*^Difference between groups was statistically significant (*P* < 0.05).

The improved FCM algorithm was applied to analyze the fMRI image data of patients, and the results are shown in Figure 2. As 5 tests were carried out, the coverages of the improved FCM algorithm were always higher than those of the preimproved HCC method. The running time of the improved FCM algorithm was only 79.8 min, while that was 113.4 min without the improvement, showing a significant difference (*P* < 0.05).

### 3.3. Effect Evaluation of PDCA Home Nursing

**Figure 3:**
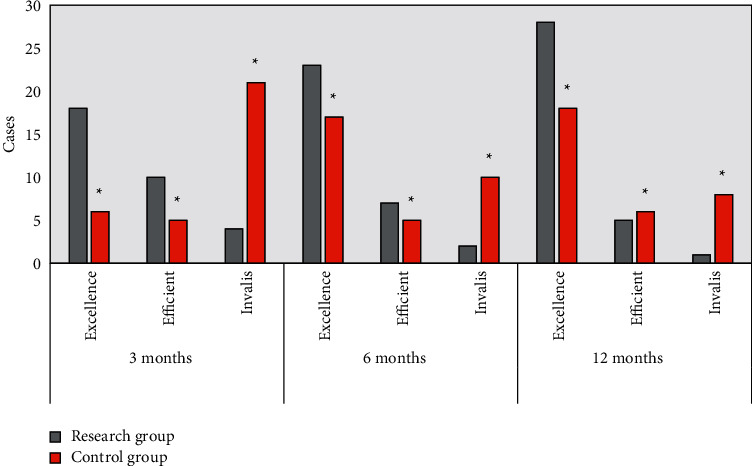
Nursing effect in different months. ^∗^Difference between groups was of statistical significance (*P* < 0.05).

The nursing effect was compared between the research group and the control group 3, 6, and 12 months after nursing, respectively. As shown in Figure 3, there was a significant difference between two groups, for the remarkedly effective rates of the research group and the control group were 56.3% vs. 18.8% in 3months, 71.9% vs. 53.1% in 6 months, and 87.5% vs. 56.3% in 12 months (*P* < 0.05). Both the remarkedly effective and effective rates pertain to the total effective rate, which was shown to be 87.5% vs. 34.4% in 3 months, 93.8% vs. 68.8% in 6 months, and 96.9% vs. 75.0% in 12 months, respectively, between the research group and the control group. The total effective rate of the research group was significantly higher than that of the control group (*P* < 0.05).

### 3.4. Evaluation of Satisfaction and Service Quality of PDCA Home Nursing

**Figure 4:**
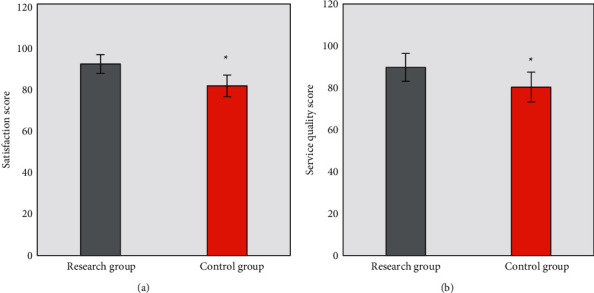
Satisfaction and service quality evaluations after nursing. (a) and (b) The scores of nursing satisfaction and the nursing service quality, respectively.  ^*∗*^Statistically significant differences between groups (*P* < 0.05).

In 12 months of nursing, the scores of nursing satisfaction and nursing service quality were compared between patients in two groups, which are shown in Figure 4. It was observed that there were significant differences in scores of nursing satisfaction (91.3 ± 4.5 vs. 80.9 ± 5.2) and the nursing service quality (89.7 ± 6.6 vs. 80.3 ± 7.1) between the two groups, and those of the research group were significantly higher than those of the control group (*P* < 0.05).

### 3.5. Evaluation of Patients' Quality of Life after Home Nursing

**Figure 5:**
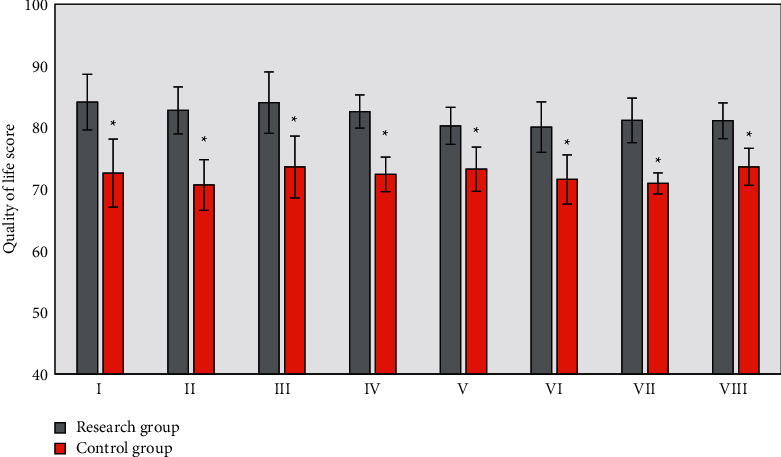
Comparison of patients' quality of life scores after nursing. I, II, III, IV, V, VI, VII, and VIII represent the general health, physical functioning, role-physical, bodily pain, social functioning, mental health, role-emotional, and vitality, respectively.  ^*∗*^Statistically significant differences between two groups (*P* < 0.05).

After 12 months of nursing, the quality of life of patients in two groups was evaluated using the SF-36 scale. As shown in Figure 5, the scores of each item of patients in the research group after nursing were significantly higher than those in the control group, with statistically significant differences (*P* < 0.05).

## 4. Discussion

DN is a chronic disease that requires long-term treatment. Only the in-hospital treatment and nursing cannot stabilize the condition of this disease in a long term. Therefore, home nursing after discharge can help patients consolidate the treatment effect and improve the quality of life [4, 13]. fMRI was used in this study to evaluate the changes in renal functions on admission and discharge. It was found that the kidney morphology of patients on discharge was fuller than that on admission, and the boundary of cortex and medulla became quite clearer on discharge. In addition, the degree of kidney fibrosis was lower, and the diffusion of water molecules was even in images. As the improved FCM algorithm was applied to detect the fMRI activation regions, the detection result was closer to the confirmed state [14]. It proved that the in-hospital treatment controlled the deterioration of DN in patients, and the improved FCM algorithm could realize the analysis and processing of fMRI image data better.

DN patients must have long-term diet control and drug treatment. The home nursing should be carried out on many aspects, such as teaching patients to self-monitor blood glucose and urine glucose, insulin injection methods, and precautions for hypoglycemic drugs. The recording is very important of proper care for the skin and the amount of edema; while the social factors should also call attention to reduce the recurrence of DN and improve the quality of life of patients [15, 16]. The innovation of this research was to evaluate the impact of fMRI under the FCM clustering algorithm on PDCA home nursing in patients with DN. For PDCA home nursing, the remarkedly effective rate of the research group and the control group was 56.3% vs. 18.8% within 3 months after nursing, 71.9% vs. 53.1% within 6 months, and 87.5% vs. 56.3% within 12 months (*P* < 0.05). PDCA nursing is a kind of planned, step-by-step, and very targeted approach, each cycle of which can make the quality of nursing improved [17]. Therefore, the effects of PDCA home nursing were analyzed and compared on the efficacy and quality of life of patients with DN after discharge from the hospital. It was shown that compared with routine home nursing, PDCA home nursing could maintain the treatment effect of patients significantly, and the scores of patients' satisfaction and service quality were significantly higher. Besides, the SF-36 scale was adopted to evaluate the quality of life of patients [18], the scores of which after PDCA home nursing were significantly higher in all dimensions than those of routine home nursing. Therefore, the PDCA home nursing could be carried out and adjusted according to the patients' personalities and disease characteristics, which was conducive to controlling the deterioration of the disease after discharge from hospital and improving the quality of life of the patients [19].

## 5. Conclusion

This study was aimed to explore the effect of fMRI under the improved FCM algorithm on PDCA home nursing for patients with DN. The improved algorithm was used to detect the activation points in the fMRI images, which gave a result closer to the real situation. Thus, it could provide assistance in diagnosis and reduce errors and misdiagnosis. At the same time, PDCA home nursing offered the great help to the recovery of patients with DN. It was superior for the consolidation of the curative effect of hospitalization, the promotion of the recovery, and the improvement of quality of life. The shortcomings of this study were that the sample size was relatively small, and the impact of nursing on patients' diabetes and renal function indicators was not taken into consideration. Therefore, the research in this field needed to be further confirmed by multicenter and large-sample clinical studies.

## Figures and Tables

**Table 1:** Comparison of patients' basic data between two groups (*n* = 64).

Basic data	Research group (*n* = 32)	Control group (*n* = 32)	Value of *P*
Age (years old)	48.3 ± 5.2	49.1 ± 6.7	0.523
Gender (male, %)	18	20	0.482
Body mass index (kg/m^2^)	23.8 ± 2.6	24.0 ± 3.1	0.544
Hemoglobin (g/L)	115.6 ± 8.9	117.2 ± 10.6	0.327
Glycosylated hemoglobin (%)	8.1 ± 1.2	7.9 ± 1.3	0.626
Cr (*μ*mL/L)	153.2 ± 20.8	155.7 ± 18.4	0.411
BUN (mmol/L)	6.7 ± 1.5	6.6 ± 5.2	0.398
eGFR (mL/min/1.73 m^2^)	71.4 ± 10.8	72.5 ± 12.6	0.339
Urine protein by creatinine ratio (mg/g)	563.2 ± 203.8	576.0 ± 182.5	0.298

eGFR, estimated glomerular filtration rate.

## Data Availability

The data used to support the findings of this study are available from the corresponding author upon request.
